# A biomimetic 3D model of hypoxia-driven cancer progression

**DOI:** 10.1038/s41598-019-48701-4

**Published:** 2019-08-22

**Authors:** Chiara Liverani, Alessandro De Vita, Silvia Minardi, Yibin Kang, Laura Mercatali, Dino Amadori, Alberto Bongiovanni, Federico La Manna, Toni Ibrahim, Ennio Tasciotti

**Affiliations:** 1 Osteoncology and Rare Tumors Center, Istituto Scientifico Romagnolo per lo Studio e la Cura dei Tumori (IRST) IRCCS, via P. Maroncelli 40, Meldola, Italy; 2 Center for Biomimetic Medicine, Houston Methodist Research Institute (HMRI), 6670 Bertner Ave, Houston, TX 77030 USA; 3 Department of Molecular Biology, Princeton University, Princeton, NJ 08544 USA; 4 Houston Methodist Orthopedics & Sports Medicine, Houston Methodist Hospital, Houston, TX 77030 USA

**Keywords:** Biological techniques, Biomimetics

## Abstract

The fate of tumors depends both on the cancer cells’ intrinsic characteristics and on the environmental conditions where the tumors reside and grow. Engineered *in vitro* models have led to significant advances in cancer research, allowing the investigation of cells in physiological environments and the study of disease mechanisms and processes with enhanced relevance. Here we present a biomimetic cancer model based on a collagen matrix synthesized through a biologically inspired process. We compared in this environment the responses of two breast tumor lineages characterized by different molecular patterns and opposite clinical behaviors: MCF-7 that belong to the luminal A subtype connected to an indolent course, and basal-like MDA-MB-231 connected to high-grade and aggressive disease. Cancer cells in the biomimetic matrix recreate a hypoxic environment that affects their growth dynamics and phenotypic features. Hypoxia induces apoptosis and the selection of aggressive cells that acquire expression signatures associated with glycolysis, angiogenesis, cell-matrix interaction, epithelial to mesenchymal transition and metastatic ability. In response to hypoxia MDA-MB-231 migrate on the collagen fibrils and undergo cellular senescence, while MCF-7 do not exhibit these behaviors. Our biomimetic model mimics the evolution of tumors with different grade of aggressiveness fostered by a hypoxic niche and provides a relevant technology to dissect the events involved in cancer progression.

## Introduction

The complex interactions between a developing tumor and the surrounding microenvironment affect the phenotype and behavior of cancer cells^1–3^ and govern processes such as progression^4,5^ and therapy response^6,7^. In recent years, engineered culture systems emerged as a unique approach to model specific physical, chemical or biological elements of the tumor microenvironment and investigate the disease mechanisms and processes with increased relevance^8^. These techniques reduced the discrepancies between *in vitro* and *in vivo* results, and allowed us to gain new insights into tumor biology^9–16^. An established cell-based example of engineered cancer models is the spheroid. Despite showing advantages over monolayer cultures, tumor spheroids lack the presence of the extracellular matrix (ECM)^17^. The ECM provides essential stimuli which affect cell function during pathophysiological events^18^. For this reason, it has been extensively used in material design for various tissue engineering applications^19,20^. In cancer, the role of the ECM within its niche is crucial and must be considered when performing functional and molecular studies or when and screening new drugs^21,22^.

Here, we present a biomimetic three-dimensional (3D) tumor model based on macroporous scaffolds obtained through a biologically inspired synthetic process, which enables the mimicking of the hierarchically organized structure of extracellular collagen^23^. Collagen is present in every tissue of the body, it generates intracellular signals by interacting with and activating cell receptors, and constitutes the necessary support for migration and proliferation^24–26^. We compared within the collagen matrix two subtypes of breast tumor cells characterized by different molecular patterns and opposite clinical outcomes: MCF-7 that belong to the luminal A subtype connected to an indolent course and good prognosis, and basal MDA-MB-231 connected to high-grade and aggressive disease^27^. Breast cancer cells in biomimetic scaffolds create a hypoxic core niche that affects multiple cell phenotypes and behaviors. We showed that hypoxia alters the cell growth dynamics, induces lineage specific responses and leads to the selection of cancer cells characterized by aggressive features, as occur in the clinical disease progression.

To our knowledge, this study provides the most comprehensive description of how hypoxia contributes to the cancer cell phenotypic evolution in lineages with different grade of aggressiveness. The possibility to model pathological hypoxia when investigating cancer biology could significantly increase the relevance and the accuracy of actual *in vitro* systems, given that this process play a key role in multiple solid tumors and hematological malignancies^28–34^.

## Results

### Cancer cells in the biomimetic scaffold create a tissue-like environment

**Figure 1:**
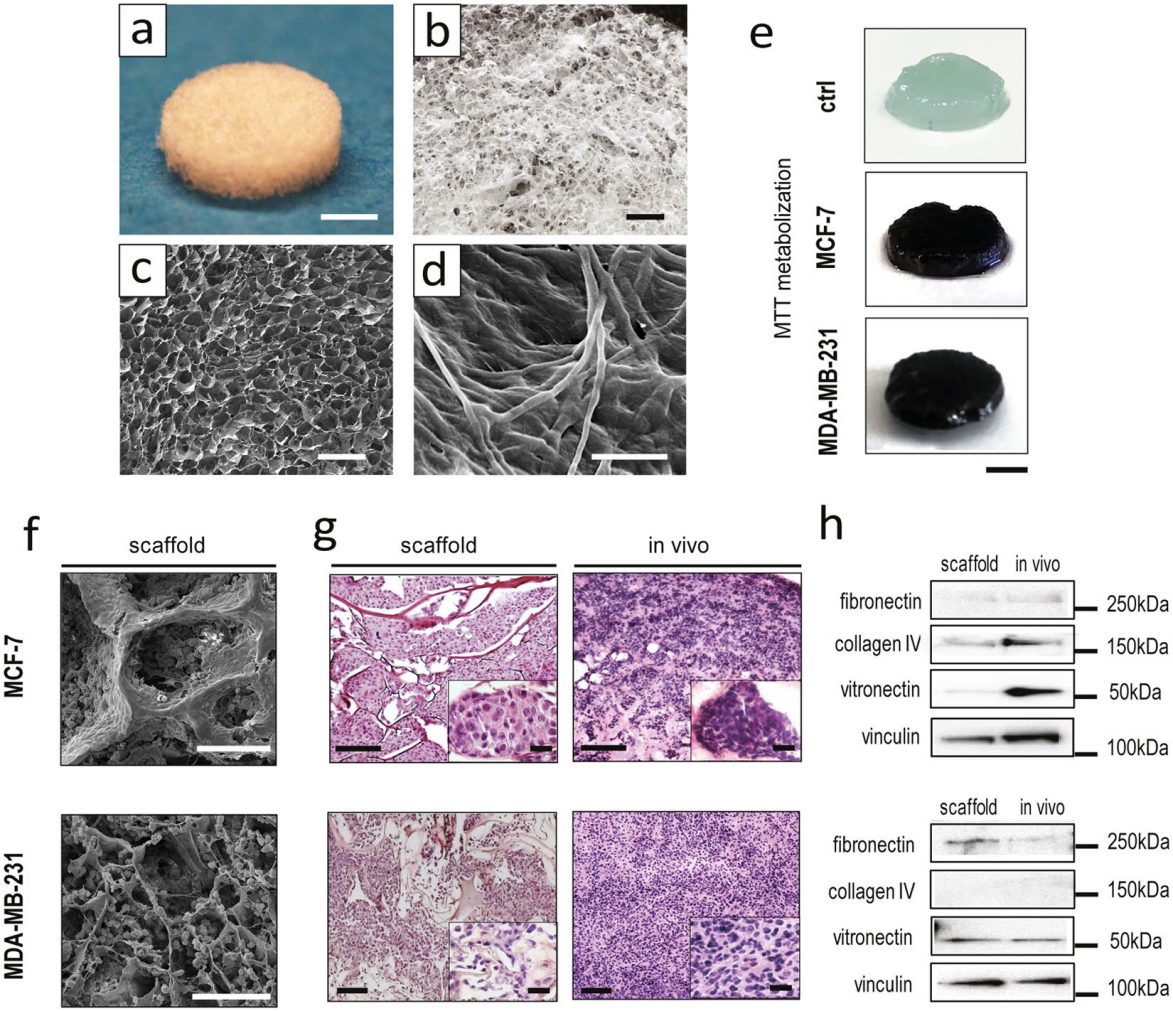
Characterization of the 3D biomimetic cancer model. (**a**,**b**) Pictures of the collagen scaffold. For a, scale bar: 3 mm. For b, scale bar: 1 mm. (**c**) SEM micrograph showing the fibrous and porous surface of the scaffold, revealing its high porosity. Scale bar: 500 µm. (**d**) High magnification SEM micrograph showing the minute architecture of the scaffold, and type I collagen fibers displaying their typical d-bands. Scale bar: 20 µm. (**e**) MTT assay of MCF-7 and MDA-MB-231 cultured on the 3D scaffold, compared to an empty control scaffold (ctrl). Scale bars: 3 mm. (**f**) SEM micrographs of MCF-7 and MDA-MB-231 cultured on the scaffold on day 7. Scale bars: 100 µm. (**g**) Hematoxylin and eosin stained histological sections of MCF-7 and MDA-MB-231 cultured on the scaffold on day 7, compared to the corresponding *in vivo* tumors. Scale bars: 100 µm. For the insets, scale bars: 20 µm. (**h**) Western blot analysis of collagen IV (180 kDa), fibronectin (285 kDa), vitronectin (54 kDa) and vinculin (125 kDa) in the scaffold and in the corresponding *in vivo* tumors.

Type I collagen scaffolds were synthesized through a pH-driven method, which enabled the fabrication of a biomimetic material with highly reproducible morphology and tunable macro- and micro-structure, as characterized by Scanning Electron Microscopy (SEM) (Fig. 1a–d and Supplementary Table S1). The collagen retained its typical D-bands organization, which was recovered during the pH-driven self-assembly (Fig. 1d).

The porous structure of the collagen matrix (average porosity of 85 ± 6.3%) allowed for cell penetration throughout the scaffold, as detected on day 10 of culture by MTT assay (Fig. 1e). After 1 week MCF-7 cells appeared flat and distributed in dense layers, while MDA-MB-231 were more globular and dispersed throughout the material’s pores (Fig. 1f). Both cell lines organized within the scaffold in a tissue-like fashion, displaying similarities with matching xenograft tumors. MCF-7 showed a round epithelial-like morphology with cells grouped in clusters, while MDA-MB-231 showed a mesenchymal-like phenotype with disorganized aspects, as reviewed in blind studies by an experienced pathologist (Fig. 1g). The protein composition of the extracellular matrix deposited by cancer cells on the scaffold recapitulated that of the corresponding xenografts, with the exception of vitronectin content that for MCF-7 was markedly higher *in vivo* (Fig. 1h).

### Cancer cells form a hypoxic core in the scaffold and activate the glycolytic pathway

**Figure 2:**
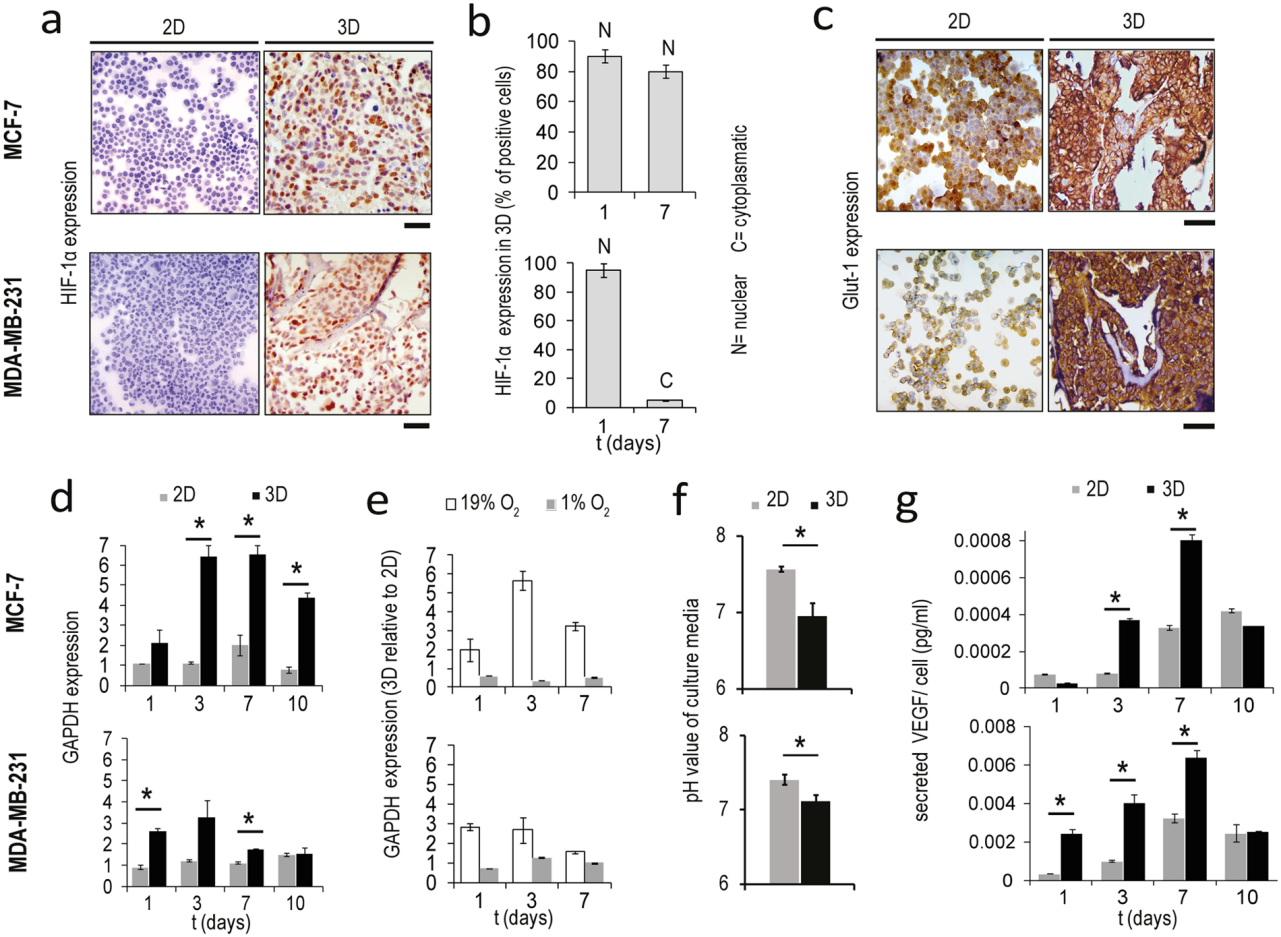
3D- cultured cancer cells show hypoxic and glycolytic characteristics. (**a**) HIF-1α expression in MCF-7 and MDA-MB-231 in monolayer culture (2D) and within the scaffold (3D) on day 1. Scale bars: 50 µm. (**b**) Percentage of MCF-7 and MDA-MB-231 positive to HIF-1α on days 1 and 7 in 3D culture. Data represent mean ± S.D. (n = 3). (**c**) Glut-1 expression in MCF-7 and MDA-MB-231 in monolayer culture and within the scaffold on day 1. Scale bars: 50 µm. (**d**) Relative expression levels of GAPDH in MCF-7 and MDA-MB-231 in monolayer culture and within the scaffold on days 1, 3, 7 and 10. Data represent mean ± S.D. (n = 3). *p < 0.05, two-tailed Student’s t-test. (**e**) Relative expression levels of GAPDH in MCF-7 and MDA-MB-231 in the scaffold versus monolayer culture on days 1, 3 and 7 in normoxic or hypoxic states (19% O_2_ or 1% O_2_ respectively). Data represent mean ± S.D. (n = 3). (**f**) Ph values of the culture media of MCF-7 and MDA-MB-231 in monolayer culture or within the 3D scaffold. Data represent mean ± S.D. (n* = *3). *p < 0.05, two-tailed Student’s t-test. (**g**) Secreted VEGF levels normalized against cell number in the culture media of MCF-7 and MDA-MB-231 in monolayer culture or within the scaffold on days 1, 3, 7 and 10. Data represent mean ± S.D. (n = 3). *p < 0.05, two-tailed Student’s t-test.

Both MCF-7 and MDA-MB-231 growing in the scaffold showed a high immunohistochemical expression of the hypoxia-inducible factor 1-alpha (HIF-1 α), while monolayer cultures proved little or no positivity (Fig. 2a). Quantification of HIF-1 α fluorescence showed that the percentage of positive spots was significantly higher in core compared to edge regions of the scaffold (for MCF7 *p* = 0.017, for MDA-MB-231 *p* = 0.005) (Supplementary Fig. S1a). This was characteristic also of xenograft tumors (for MCF7 *p* = 0.005). The presence of a hypoxic niche was confirmed by pimonidazole staining, which permitted the visualization of poorly oxygenated cells in the scaffold and xenograft sections (Supplementary Fig. S1b). The two lineages demonstrated a different temporal response to hypoxia: while MCF-7 exhibited high HIF-1α expression with nuclear localization for all culture time, the percentage of MDA-MB-231 positive for HIF-1α decreased from 90–95% on day 3 to 5% on day 7, concomitantly with the shift of the protein localization from nuclear to cytoplasmic (Fig. 2b). As an adaptation to low-oxygen environments, cancer cells predominantly produce energy by glycolysis^35^. Both MCF-7 and MDA-MB-231 displayed within the scaffold a high immunohistochemical expression of the glucose transporter 1(Glut-1) (Fig. 2c) and a significant upregulation of the mRNA level of the glycolysis enzyme glyceraldehyde 3-phosphate dehydrogenase (GAPDH) compared to monolayer cells (for MCF7 *p* = 0.006, *p* = 0.0011 and *p* = 0.003 on day 3, 7 and 10, respectively; for MDA-MB-231 *p* = 0.007 and *p* = 0.005 on day 1 and 7, respectively) (Fig. 2d). The temporal expression of GAPDH correlated with the hypoxic profile: MCF-7 showed a high and constant upregulation of this marker, while MDA-MB-231 showed lower levels of GAPDH induction with a decrement on days 7 and 10. To verify the correlation between expression of GAPDH and oxygen concentration, we next cultured cells under 1% O_2_. In hypoxic state the expression of this marker was significantly enhanced (Supplementary Fig. S1c), while expression differences between 2D and 3D cultures were reduced (Fig. 2e). This finding suggests that the upregulation of GAPDH observed in the scaffold under normoxic state was mediated by 3D-induced hypoxia. As a result of impaired cellular respiration, the pH levels in the tumor microenvironment are often lower than those of normal tissues. After 4 days of culture we observed a significant decrease in the pH value of the scaffold media compared to that of monolayer culture, with a decrement of 0.6 pH units in MCF-7 and of 0.3 pH units in MDA-MB-231(*p* = 0.0003 and *p* = 0.0012, respectively) (Fig. 2f). Finally, we analyzed the secretion of the pro-angiogenic factor VEGF in the culture media, as hypoxia is one of the major drivers of tumor-induced angiogenesis^33^. A significant increase in VEGF levels was found in the scaffold media compared to monolayer culture for both cell lines (for MCF7 *p* = 0.002 and *p* = 0.006 on day 3 and 7, respectively; for MDA-MB-231 *p* = 0.004, *p* = 0.01 and *p* = 0.008 on day 1, 3 and 7, respectively) (Fig. 2g). Notably, MDA-MB-231 secreted about 10 times more VEGF than MCF-7.

### Cell growth dynamics in the scaffold are characterized by constant proliferation and induction of apoptosis

**Figure 3:**
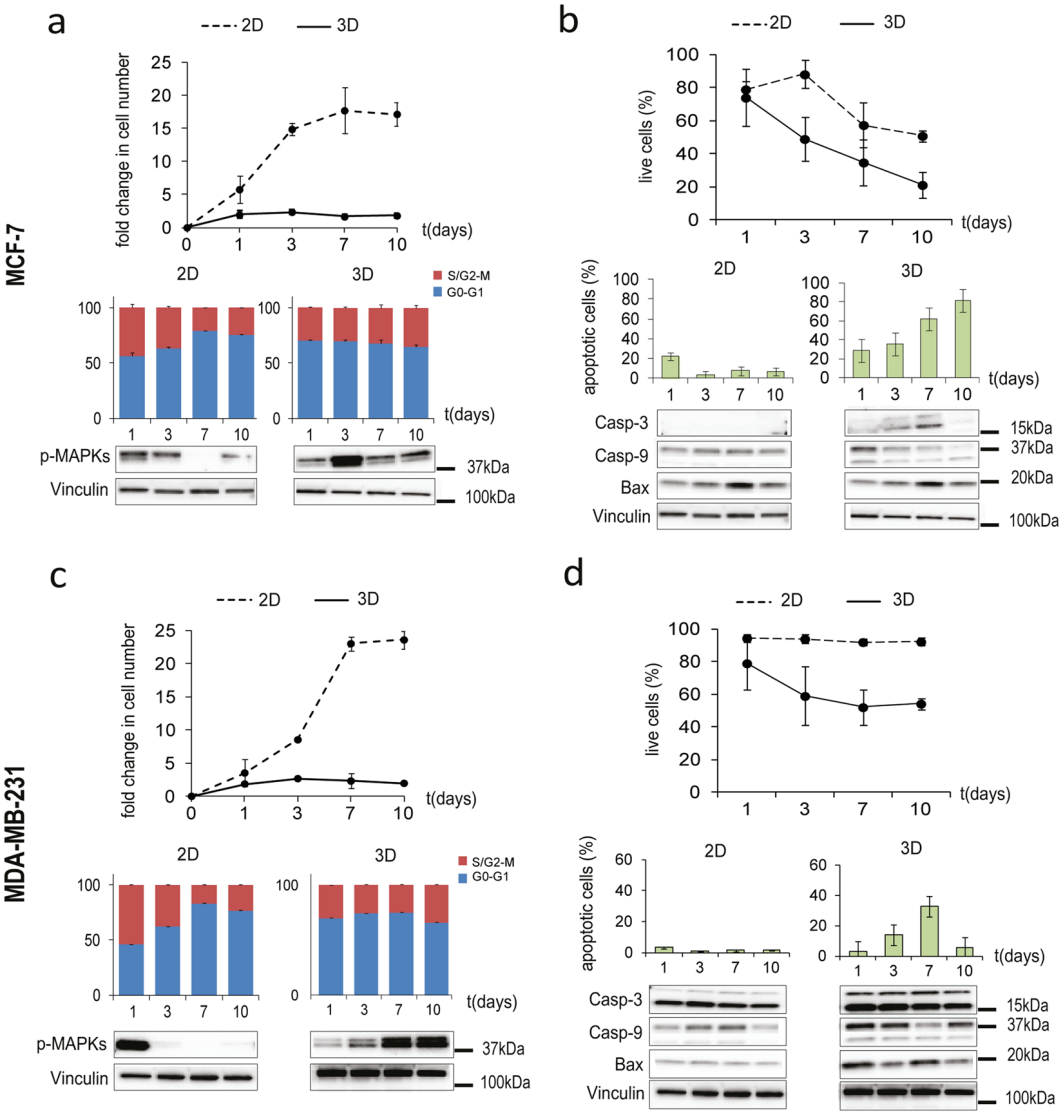
Cancer cell growth dynamics in the 3D biomimetic model. (**a**,**c**) Fold changes in cell number (relative to day 0) for MCF-7 and MDA-MB-231 in monolayer culture (2D) or within the scaffold (3D) on days 1, 3,7 and 10; percentages of cells in S and G2/M phases or in G0/G1 phases; western blot for p-MAPK (44, 42 kDa). Data represent mean ± S.D. (n = 3). (**b**,**d**) Percentages of live cells for MCF-7 and MDA-MB-231 in monolayer culture (2D) or within the scaffold (3D) on days 1, 3,7 and 10; percentages of apoptotic cells; western blot for Caspase-3 (19, 17 kDa), Caspase-9 (37, 35 kDa) and Bax (20 kDa). Data represent mean ± S.D. (n = 3).

Cancer cells within the scaffold demonstrated a slower rate of proliferation than in monolayer culture, but constant over time, as showed by the fold changes in cell number and by the percentages of cells engaged in the S and G_2_/M cycle phases (Fig. 3a,c). Conversely, in monolayer culture cells showed typical proliferation dynamics characterized by an exponential growing phase followed by a confluence-induced cycle arrest with a progressive decrease in the S and G_2_/M populations in favor of an increase of the G_0_-G_1_ population. Moreover, phosphorylation of the mitogen-activated protein kinases (MAPKs) Erk1 and Erk2 decreased or was lost over time in monolayer cells, whereas cells within the scaffold showed activation of these proteins for the entire duration of the culture, with a peak on day 3 for MCF7 and on days 7 and 10 for MDA-MB-231. The dynamic of growth occurring in the 3D model reproduced more closely that of a developing *in vivo* tumor mass: the fold changes in cell number matched with the fold changes in xenograft volume (Supplementary Fig. S2a). We next observed a marked decrease over time in the percentage of live cells within the scaffold, parallel to an increasing number of apoptotic events (Fig. 3b,d). Conversely, in monolayer cultures viability decreased to a lesser extent in MCF-7 (Fig. 3b), and was stable at around 95% in MDA-MB-231 (Fig. 3d), and few apoptotic cells were detected. *In-situ* TUNEL staining confirmed the increase of apoptotic cells in 3D culture compared to monolayer, with percentages matching those of flow cytometry analysis at all time points (days 1,3,7 and 10) and culture conditions (Supplementary Fig. S2b). Consistently, both cell lines in the scaffold expressed higher levels of the Caspase-3 and 9, their cleaved activation fragments, and the Bcl-2-associated X protein (Bax) compared to monolayer cultures (Fig. 3b,d). The presence of apoptotic regions, defined by low cellularity and extensive chromatin condensation, was confirmed in hematoxylin and eosin (H&E) stained histological sections of the corresponding *in vivo* tumors (Supplementary Fig. S2c), and by *in-situ* TUNEL-positivity (Supplementary Fig. S2d). The viability and apoptosis profiles matched the kinetic of HIF-1α and GAPDH expression in both lineage, providing a correlation between hypoxia and growth dynamics: while MCF-7 displayed a constant decrease in viability parallel to the increase in apoptotic cells (Fig. 3b), for MDA-MB-231 no significant changes in viability were observed after day 3, and the percentage of apoptotic cells decreased after day 7 (Fig. 3d).

### Cancer cells in the scaffold evolve to a more aggressive phenotype

**Figure 4:**
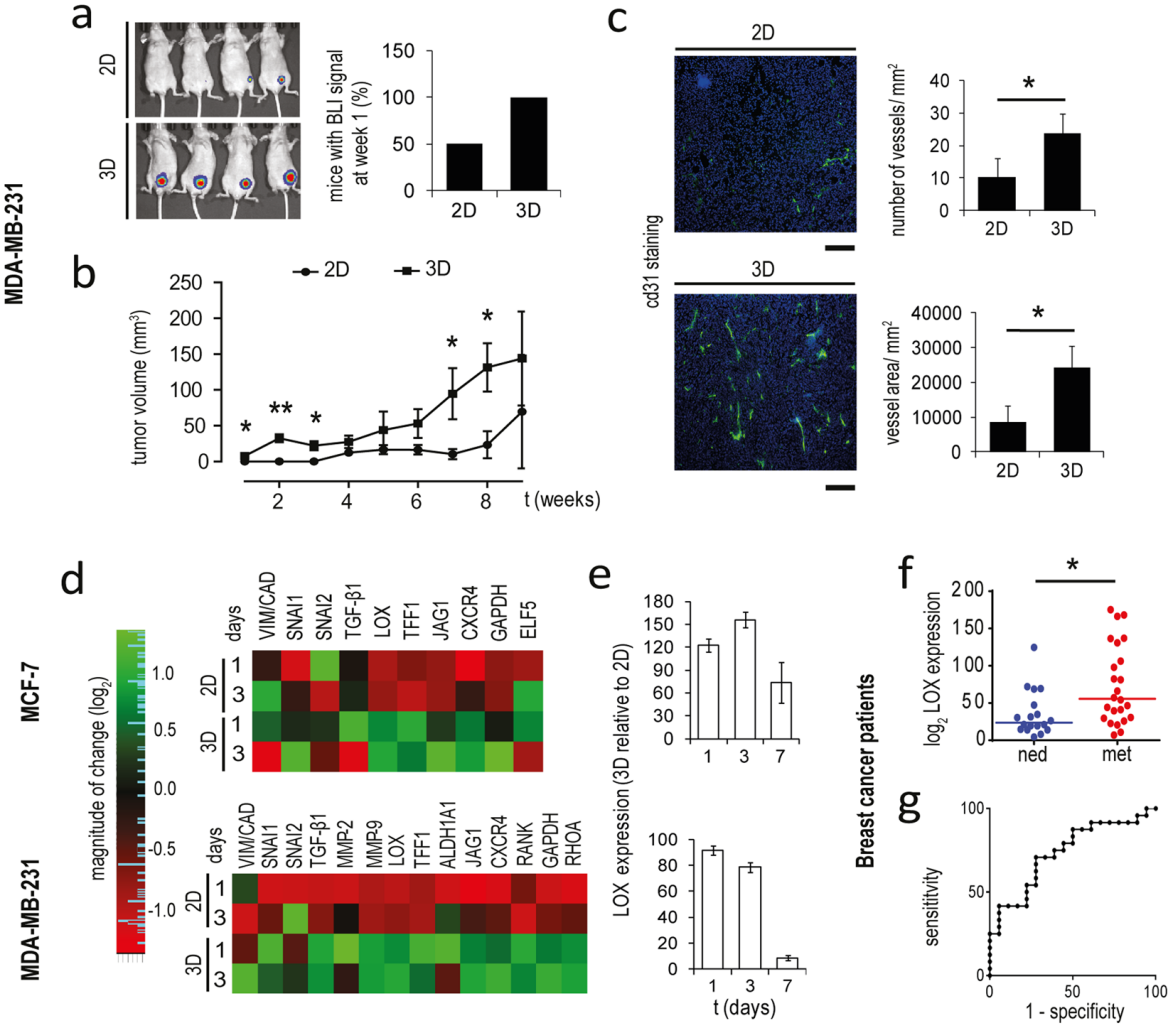
Cells cultured in the scaffold exhibit aggressive features. (**a**) Whole body IVIS imaging and percentage of mice with detectable tumors after 1 week from the orthotopic injection of MDA-MB-231 cultured in monolayer (2D) or in the scaffold (3D). (**b**) Tumor volume of mice with orthotopic injection of 2D and 3D cultured MDA-MB-231. Data represent mean ± S.E.M (n = 5) *p < 0.05. (**c**) Representative images of Cd31 stained histological sections, number of vessel for mm^2^, and vessel area for mm^2^ in xenograft tumors generated by orthotopic injection of 2D and 3D cultured MDA-MB-231. Cd31 positive cells (green) and nuclei stained with DAPI (blue). Scale bars: 300 µm. Data represent mean ± S.D. (n* = *3). (**d**) Heatmap visualization of relative expression values of biomarkers related to tumor aggressiveness in MCF-7 and MDA-MB-231 in monolayer culture or within the scaffold. (**e**) Relative expression levels of LOX in MCF-7 and MDA-MB-231 within the scaffold versus monolayer cultures on days 1, 3 and 7. (**f**) Relative expression levels of LOX in breast cancer patients. *p < 0.01, two-sample Wilcoxon rank-sum test. (**g**) ROC curve of LOX accuracy as prognostic factor in patients with breast cancer.

We compared the *in vivo* tumorigenic potential of monolayer and scaffold cultured MDA-MB-231-*Luc2* in an orthotopic mouse model. Cells from the scaffold formed detectable tumors much faster: the 100% of mice displayed BLI signals 1 week after implantation, compared to the 50% of the mice injected with monolayer cells (Fig. 4a). The growth rate was also significantly higher (*p* = 0.027, *p* = 0.001, *p* = 0.012, *p* = 0.049 and *p* = 0.041 at week 1, 2, 3, 7 and 8, respectively, Fig. 4b) and tumors showed an increased vascularization, both in term of vessel number and area fraction (*p* = 0.013 and *p* = 0.008, respectively) (Fig. 4c). Consistently with the enhanced *in vivo* aggressiveness, MDA-MB-231 in the scaffold exhibited a higher Vimentin to Cadherin E ratio, and a significant upregulation of the matrix modifying enzymes MMP-2, MMP-9 and LOX, the chemokine receptor CXCR4, the bone metastasization markers RANK and JAG-1, the stemness marker ALDH1A1, the contractility mediator RHOA, the EMT regulators TGF-β1, SNAIL, SLUG and the oncogene TFF1 compared to cells in monolayer (Fig. 4d). MCF-7 showed the upregulation of LOX, TFF1, CXCR4, JAG-1 and to a lesser extent of TGF-β1 and SNAIL. With exception of SNAIL for which a direct regulation by collagen binding is known^36^, the differences between 2D and 3D settings decreased when cells were cultured under 1% O_2_, underlying the correlation of hypoxia with the induction of these biomarkers (Supplementary Fig. S3a).

### Lysyl oxidase has prognostic significance in breast cancer patients

For both cell lines LOX was the mostly upregulated marker in the scaffold model: 3D-expression levels reached about 155 and 90 times those of cells in monolayers, for MCF-7 and MDA-MB-231 respectively (Fig. 4e). LOX induction was linked to hypoxic signaling as culturing under 1% O_2_ significantly enhanced the expression of this marker (Supplementary Fig. S3b) and reduced the differences between the two culture conditions (Supplementary Fig. S3c). Moreover, LOX expression in MDA-MB-231 *in vivo* was positively associated with higher tumor volumes (Supplementary Fig. S3d). We thus hypothesized that LOX is involved in the progression of primary breast tumors, and analyzed the prognostic significance of its expression in a cohort of 41 human breast cancer patients. LOX expression levels correlated with disease aggressiveness, being significantly higher in the primary tumors of relapsed patients over patients with no evidence of disease (NED) after a 10 year follow up (*p* = 0.0088). The median expression value was 55.7 for patient that developed metastatic disease (n = 22), while it was 26.3 for NED ones (n = 19) (Fig. 4f). The receiver operating characteristics (ROC) curve to predict relapse for LOX levels indicated a good prognostic accuracy with an area under the curve (AUC) of 0.74 (Fig. 4g).

### MDA-MB-231 migrate on collagen fibrils in response to hypoxia

**Figure 5:**
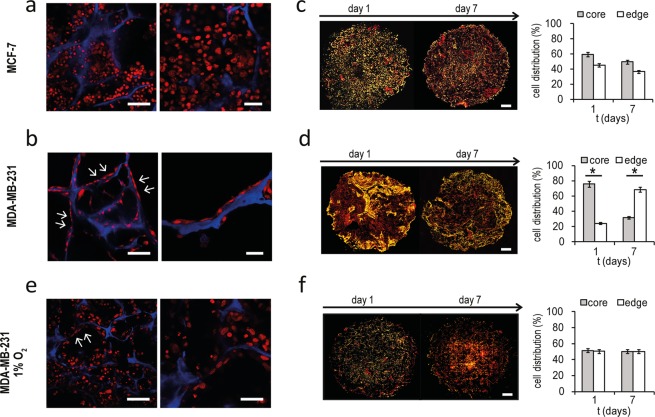
MDA-MB-231 migrate at the scaffold edges over time. (**a**,**b**) Confocal microscopy images at different magnifications of MCF-7 and MDA-MB-231 within the scaffold on day 7. Cells are stained with DRAQ5 (red) and blue is the collagen scaffold autofluorescence. For the left panels, scale bars: 50 µm. For the right panels, scale bars: 20 µm. (**c**,**d**) Whole images of histological sections of scaffold cultured with MCF-7 and MDA-MB-231 on day 1 and 7, and percentages of cells in edge or core regions of the scaffold. Cells are stained with DAPI (yellow) and red is the collagen scaffold autofluorescence. Scale bars: 1 mm. Data represent mean ± S.E.M (n = 3). (**e**) Confocal microscopy images of MDA-MB-231 within the 3D scaffold on day 7 under hypoxic state (1% O_2_). Cells are stained with DRAQ5 (red) and blue is the collagen scaffold autofluorescence. For the left panel, scale bar: 50 µm. For the right panel, scale bar: 20 µm. (**f**) Whole images of histological sections of scaffold cultured with MDA-MB-231 on day 1 and 7 under hypoxic state, and percentages of cells in edge or core regions of the scaffold. Scale bars: 1 mm. Data represent mean ± S.E.M (n = 3). Cells are stained with DAPI (yellow) and red is the collagen scaffold autofluorescence.

While MCF-7 are epithelial-like cells associated with a weak invasiveness, MDA-MB-231 are enriched for epithelial to mesenchymal transition (EMT) markers, and possess higher phenotypic plasticity and a more invasive behavior^27^. We previously observed that MDA-MB-231 after 3 days of culture reduced the expression of hypoxic and glycolytic signals and displayed a constant viability, while MCF-7 showed a progressive decrease in the number of live cells and a stable expression over time of the markers of hypoxia and glycolysis. Confocal microscopy analysis of the scaffolds on day 7 showed that MCF-7 aggregated in clusters with an epithelial-like morphology (Fig. 5a). Conversely, MDA-MB-231 cells displayed a spindle-shaped mesenchymal phenotype, reduced cell density and an aligned distribution (arrowhead) over the collagen fibrils, resembling the invasive morphology of lobular breast tumors described as “Indian filing”^37^ (Fig. 5b). DAPI-stained images of whole scaffold sections demonstrated that MDA-MB-231 concentrated at peripheral regions over time: the percentage of cells at the scaffold edges increased from 20% on day 1 to 70% on day 7 (Fig. 5d and Supplementary Fig. S4a). Conversely, for MCF-7 the cell distribution in core and edge areas was similar between day 1 and day 7 (Fig. 5c and Supplementary Fig. S4a). This result and the Indian file pattern suggest that MDA-MB-231 migrated toward the scaffold’s peripheral regions over time. Activation of migratory signaling was confirmed by the protein expression of Rho GTPase, a cytoskeleton contractility mediator involved in cellular motility. Rho was markedly induced in MDA-MB-231 cultured within the scaffold compared to cells in monolayer culture (Supplementary Fig. S4b), while it was not affected in MCF-7. We next performed analysis of cell morphology and distribution under 1% oxygen pressure. In a hypoxic state we found MDA-MB-231 with enhanced cell-cell contact, the Indian filing pattern (arrowhead) partially reverted (Fig. 5e) and uniform cell distribution across scaffold regions (Fig. 5f). These findings indicate that under experimental normoxic conditions, migration of MDA-MB-231 was fostered by the gradient between a more hypoxic core and well oxygenated edges, which was lost in 1% O_2_. The ability of these cells to concentrate at the scaffold’s peripheral regions correlate with the decrease in apoptotic, hypoxic and glycolytic signals observed on day 7 of culture. Conversely, exposure of MCF-7 to 1% O_2_ did not result in a different cell distribution compared to normoxic state (Supplementary Fig. S4c). Finally, in several areas of *in vivo* MDA-MB-231 tumors we identified cells aligned in Indian file, demonstrating that this migratory pattern might be a distinctive feature of invasive breast cancer (Supplementary Fig. S4d). Interestingly, Indian file regions (arrowhead) were observed in close proximity to CD31 positive tumor vessels (Supplementary Fig. S4d).

### Hypoxia induces cellular senescence in MDA-MB-231

**Figure 6:**
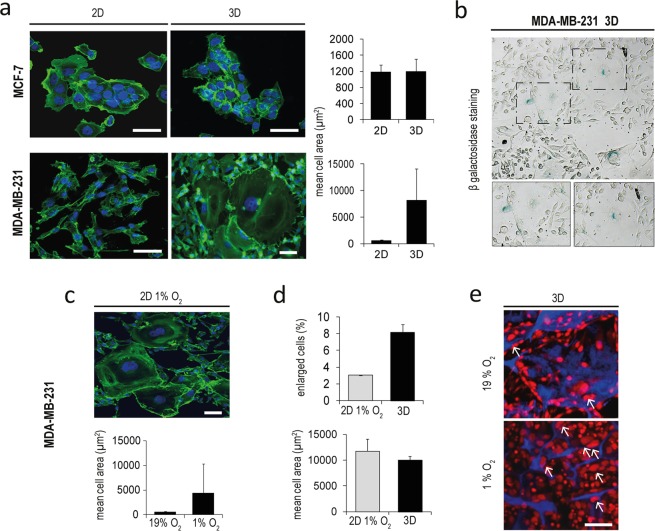
Hypoxia induce senescence in MDA-MB-231. (**a**) Inverted fluorescent microscope images and mean cell area (µm^2^) of MCF-7 and MDA-MB-231 in monolayer cultures (2D) or recovered after 7 days in the scaffold (3D). Staining for F-actin (phallodin, green) and nuclei stained with DAPI (blue). Scale bars: 20 µm. (**b**) Bright field representative images of cells positive for β-galactosidase staining (blue) in MDA-MB-231 recovered after 7 days within the 3D scaffold at different magnification. (**c**) Inverted microscope images of MDA-MB-231 in monolayer cultures (2D) under hypoxic (1% O_2_) state; mean cell area of MDA-MB-231 in monolayer cultures under normoxic (19% O_2_) or hypoxic (1% O_2_) state. Scale bar: 20 µm. Staining for F-actin (phallodin, green) and nuclei stained with DAPI (blue). (**d**) Percentage of MDA-MB-231 with senescent phenotype and mean cell area after 7 days of hypoxic monolayer culture (2D 1% O_2_) or within the scaffold (3D). (**e**) Confocal microscopy images of MDA-MB-231 cultured within the scaffold on day 7 in normoxic or hypoxic states (19% O_2_ or 1% O_2_ respectively). Cells are stained with DRAQ5 (red) and blue is the collagen scaffold autofluorescence. Arrow indicate cells with enlarged dimension. Scale bars: 50 µm.

Senescence is a growth-arrest program defined by morphological alterations such as enlarged and flat cytoplasm and the expression of the β-galactosidase enzyme, which can occur as a result of different external stresses^38^. MDA-MB-231 recovered after 7 days within the scaffold exhibited a marked increase in cellular dimensions with respect to cells in monolayer (Fig. 6a). The mean cell area ranged from 618 µm^2^ in monolayer to 8,212 µm^2^ in cells from the scaffold, whereas mean cell area of MCF-7 was not affected (Fig. 6a). Morphologically enlarged MDA-MB-231 proved positive for the expression of the senescence-associated β-galactosidase marker (Fig. 6b). To confirm the phenotype we performed morphology evaluation directly on cells cultured within the scaffold. MCF7 cells were mostly round with regular shapes. The dimension of the cytoplasm was constant, as observed in recovered cells. MDA-MB-231 showed a markedly variable morphology, with rounded cells, elongated cells and cells with enlarged nucleus (arrowhead) and cytoplasm (Supplementary Fig. S5). MDA-MB-231 in monolayer culture under 1% O_2_ showed similar senescence-linked morphological alterations, as proof that this process was related to the hypoxic condition (Fig. 6c). The percentages of senescent cells over the total population was 8% and 3% with a mean cell area of 9,976 µm^2^ and 11,753 µm^2^, respectively for cells in the scaffold or in hypoxic monolayer culture (Fig. 6d). Moreover, a strong increase in the number of enlarged cancer cells (arrowhead) was observed in MDA-MB-231within the scaffold when cultured under hypoxia, as determined by confocal microscopy (Fig. 6e). The presence of β-galactosidase positive cells was found also in histological sections of *in vivo* MDA-MB-231 tumors (Supplementary Fig. S6a). As observed for HIF-1α, the percentage of β-galactosidase positive spots was markedly higher in core regions. The presence of senescence in MDA-MB-231 cultured within the scaffold, growing *in vivo* and in monolayer under hypoxic state was further confirmed by direct lipofuscin staining (Supplementary Fig. S6b).

## Discussion

Tissue engineered models provide the ideal tool to investigate cells in native environmental conditions allowing for the preservation of their phenotypes, genotypes and behavior while offering high-throughput analyses and cost-efficient screenings^39–42^. Here we developed a 3D technology based on collagen scaffolds that enables the modeling of the tumor hypoxic environment and its contribution in the cancer cell evolution. We demonstrated that the use of a biomimetic 3D environment results in cancer cells showing a pathological hypoxic state. Hypoxia affects cell growth dynamics and induces the acquisition of aggressive features. Different signaling pathways were found to be associated with the hypoxia-driven phenotype: cells acquire markers of glycolysis, angiogenesis, cell-matrix interaction, migration, EMT and metastatic ability (Supplementary Fig. S7). Profiling of cancer cells within this model could foster the discovery of novel prognostic factors involved in the clinical progression of solid tumors, while these pathways are normally inactive or altered in monolayer cultures. We provided evidence for the validation of this hypothesis in human patient samples. We demonstrated that expression of LOX, the gene with the highest level of induction in biomimetic scaffolds, has prognostic value in breast cancer patients, being significantly higher in the primary tumor of patients that developed metastatic disease. In agreement with our results, a previous study had highlighted the role of LOX in the formation of pre-metastatic niches at bone site in ER^−^ breast tumors^43^. Of note, LOX was recently found to be a poor prognostic indicator in various solid tumors such as non-small cell lung cancer, nasopharyngeal carcinoma and prostate cancer^44–46^.

Moreover, we showed that tumor subtypes with different grade of aggressiveness display distinct responses to hypoxia and phenotypic evolution. As a result of the core low oxygenation, MDA-MB-231 were found to be concentrated at the edges of the scaffold, a possible explanation for which could be the activation of a migratory process toward regions with higher oxygen levels. This process mimics *in vivo* tissue invasion and permits to cancer cells to reach favorable niches and increase cell viability, while epithelial MCF-7 do not display this behavior (Supplementary Fig. S7). MDA-MB-231 also respond to hypoxic conditions by undergoing senescence. This is an interesting observation as evidence has suggested that senescence may drive different tumor promoting processes. In particular, the senescence-associated secretory phenotype (SASP) has been found to sustain the malignant progression of solid tumors, inducing cancer stemness^47^ and acting as a relapse-promoting factor in tumor treatment^48^. There is also evidence that senescent cells may facilitate the microenvironment modulation and promote cancer cell invasion^49^. Our results highlighted that senescence is induced by hypoxia in cancer cells displaying an aggressive phenotype. This model could be used to study whether senescence has a protective role in the response of cancer cells to hypoxic stress, and whether this process impacts the cell invasive phenotype. Furthermore, the possibility of identifying differential behaviors between highly and weakly aggressive cancer cells could be critically important when investigating cancer primary cultures.

Through the comparison with the corresponding xenotransplants we have also demonstrated that the pathological phenotypes and behaviors recapitulated in this biomimetic model match with those of *in vivo* growing tumors. From the technical standpoint the use of collagen as the main component of the scaffold will allow for the evolvement of our system into progressively complex models as: (i) it is the main ECM protein of every tissue of the body and its use can be extended to culture other types of solid tumors^26^; (ii) the possibility to create complex geometries and intra-scaffold compartments and the abundance of functional groups enable for the introduction of other ECM components, bioactive factors and stromal cells to engineer tissue-specific tumor niches. This model also provides broad applicability as the large pore size enables the easy cellularization of the material without the need for a bioreactor, and the system allows for viable cell recovery and compatible with high-throughput downstream analysis^50,51^. Compared to previously reported 3D systems^11,13,16,17^, our model encompasses multiple hypoxia-driven cell behaviors and phenotypes, each reflecting key characteristics of *in vivo* growing tumors. Our systematic description of the interplay connecting the observed phenotypes, and their role in cancer cell growth and evolution open up new possibilities for investigating different mechanisms and processes that are not represented in current culture models. The limitation of our system was the use of breast cancer as a single model. The study of other cell lineages would help us to understand how signaling pathways generated in collagen scaffolds affect the growth and evolution of other types of solid tumors, thus providing a unique opportunity to elucidate the role of collagen in cancer progression where it has demonstrated a crucial but controversial function^52^.

In conclusion, our scaffold models the evolution of solid tumors fostered by a hypoxic niche and allows for the investigation of unexplored events and mechanisms involved in cancer progression and in the onset of aggressive cancer behaviors.

## Materials and Methods

### Collagen scaffold synthesis

All chemicals were purchased from Sigma Aldrich (St. Louis, MO, USA). The collagen scaffolds were synthesized as previously described^23,50^. Briefly, a 1 wt% suspension of type I collagen in acetic acid was prepared and precipitated to pH 5.5. The material was cross-linked through a 1 wt% 1, 4-butanediol diglycidyl eter (BDDGE) to stabilize the collagen matrix and to control porosity and tortuosity. Scaffold’s porosity and pore size was obtained through an optimized freeze-drying process, consisting of an established freezing and heating ramp (from 25 °C to −25 °C and from −25 °C to 25 °C in 50 min under vacuum conditions, *p* = 0.20 mbar), ultimately ensuring proper pore interconnectivity and orientation. All scaffolds were sterilized by immersion in 70% ethanol for 1 hour, followed by 3 washes in sterile Dulbecco Phosphate Buffered Saline (DPBS) (Life Technologies, Carlsbad, CA, USA).

The porosity of the scaffold was determined through an ethanol infiltration method as previously described^23,50^. Pore size was measured by the Nova NanoSEM™ SEM software.

### Cell seeding and culture

The experiments were performed on two human breast cancer cell lines, MDA-MB-231 and MCF-7, obtained from the America Type Culture Collection (Rockville, Maryland, USA). All cells were maintained in DMEM medium supplemented with 10% fetal bovine serum, 1% penicillin-streptomycin and 1% glutamine (PAA, Piscataway, NJ, USA) at 37 °C in a 5% CO_2_ atmosphere. For standard cultures, 6 × 10^5^ cells were maintained as a monolayer in 25-cm^2^ flasks in 3 ml of culture media. For 3D cultures, each scaffold (1 × 9 mm) was placed in a 6-multiwell plate and seeded with 5 × 10^6^ cells by adding 50 µl of cell suspension on the scaffold upper surface. Seeding was reached by simple soaking of the cell suspension in the dry scaffolds. Cells were incubated for 1 hour at 37 °C to allow adhesion, after that 4 ml of culture medium were carefully added. After 24 h, the scaffolds were gently moved in a new 6-multiwell plate to avoid any contribution of cells that might have attached on the plate surfaces. The medium was replaced daily. Cultures under hypoxic conditions were performed in a hypoxic workstation (Ruskinn Technology, Bridgend, UK) with continuous 1% O_2_ levels. MDA-MB-231 and MCF-7 cells marked with luciferase for the *in vivo* study were maintained in selection media [DMEM high glucose (4.5 g/l) with 10% FCII (Fetal Clone II, Hyclone), 1% penicillin/streptomycin, 1% glutamine without sodium pyruvate and 800 µg/ml of Geneticin (G418 Invitrogen for selection of luciferase)] and cultured as previously described.

### SEM and confocal microscopy

Cells seeded in 3D collagen scaffolds were imaged by SEM and Laser Confocal Microscopy as previously described^50^. For SEM imaging samples were washed 3 times with 0.1 M sodium cacodylate buffer pH 7.4, fixed in 2.5% glutaraldehyde in 0.1 M sodium cacodylate buffer pH 7.4 for 2 h at 4 °C and washed again in 0.1 M sodium cacodylate buffer pH 7.4 (Sigma Aldrich). Samples were then dehydrated in a graded series of ethanol for 10 min each, dried in a dessicator overnight and sputter-coated with platinum. Images were acquired with a Nova NanoSEM 230 scanning electron microscope (FEI, Hillsboro, OR, USA). For confocal imaging, cells were washed 3 times with 1% PBS, fixed with 4% paraformaldehyde for 20 minutes at room temperature and stained with 10 µM/ml DRAQ5™ (ImmunoChemistry Technology, Bloomington, MN, USA). Images were acquired with an A1 laser confocal microscope (Nikon Corporation, Tokyo, Japan) and analyzed with the NIS Elements software (Nikon Corporation, Tokyo, Japan).

### Cell proliferation analysis

Cell proliferation in 3D scaffolds or in monolayer cultures was assayed by total DNA content quantification using the PicoGreen dsDNA assay (Invitrogen, Carlsbad, CA, USA). Briefly, total DNA was extracted using DNeasy Blood & Tissue Kit (Qiagen, Duesseldorf, Germany) following the manufacturer’s instructions. 100 µL of supernatant was then added to 100 µL of PicoGreen reagent working solution in a 96-well plate. Fluorescence of the samples was measured with a microplate reader (FLUOstar OPTIMA BMG LABTECH, Ortenberg, Germany) with excitation and emission wavelengths of 480 nm and 520 nm, respectively. The exact number of cells was calculated from the total DNA content using the conversion factor of 7.7 pg DNA per cell.

### MTT assay

Briefly, cells within the scaffolds or in monolayer cultures were incubated with 0.5 mg/ml of MTT solution (Sigma Aldrich) in DMEM for 2 hours at 37 °C and the absorbance was determined at 550 nm.

### Quantitative real-time reverse transcriptional-PCR (qRT-PCR)

RNA extraction and qPCR were performed as previously described^53^. The scaffolds were fragmented into small pieces, while 2D culture cells were collected by tripsinization. Total mRNA was isolated using TRIzol Reagent (Invitrogen) following the manufacturer’s instructions. Five hundred nanograms of RNA were reverse-transcribed using the iScript cDNA Synthesis Kit (BioRad, Hercules, CA, USA). The final mixture was incubated at 25 °C for 5 min, at 42 °C for 20 min, at 47 °C for 20 min, at 50 °C for 15 min and 5 min at 85 °C. Real-Time PCR was performed on the 7500 Real-Time PCR System (Applied Biosystems, Foster City, CA, USA) using the TaqMan gene expression assay mix (Applied Biosystems). Amplification was performed in a final volume of 20 µl containing 2x Gene expression master Mix (Applied Biosystems), 2 µl of cDNA in a total volume of 20 µl. The reaction mixtures were all subjected to 2 min at 50 °C, 10 min at 95 °C followed by 40 PCR cycles at 95 °C for 15 sec and 60 °C for 1 min for overall markers. The stably expressed endogenous β-actin and HPRT were used as reference genes. Nineteen markers were analyzed: MMP-2, MMP-9, RHOA, HRAS, VIM, CDH1, LOX, TGF-β1, CXCR-4, SNAI1, SNAI2, ALDH1A1, CTNNB1, GAPDH, ELF5, TFF1, RANK, RANK-L and JAG1. The amount of transcripts was normalized to the endogenous reference genes and expressed as n-fold mRNA levels relative to a calibrator using a comparative threshold cycle (Ct) value method (ΔΔCt). For all analysis, the calibrator used was a mix of the RNA extracted from MCF7 and MDA-MB-231 cultured in 2D for 24 h.

The heatmap visualization and level plots of gene expression data were generated through R software (R Core Team (2015). R: A language and environment for statistical computing. R Foundation for Statistical Computing, Vienna, Austria URL: https://www.R-project.org.

### Retrospective patient study

LOX expression levels were retrospectively evaluated in fresh frozen primary tissues of 41 female breast cancer patients enrolled from 1997 to 2000. Patients aged ≥18 years with histologically confirmed breast cancer who underwent radical surgery were eligible. Patients might have received adjuvant therapy (chemotherapy or hormone therapy according to ER/PgR and HER-2 status). All patients were followed for a minimum of 10 years. Twenty-two patients developed metastatic disease (MET) during the follow-up period, while nineteen patients had no evidence of disease (NED) at the time of the last follow up.

### Cell recovery from scaffolds and morphological evaluation

To recover cells from the 3D constructs, the scaffolds were disaggregated into 1–2 mm^3^ pieces with sterile surgical blades and enzymatically digested in 2 mg/ml type I collagenase (Merck Millipore, Darmstadt, Germany) for 1 h at 37 °C in stirring conditions. Cell suspension was then filtered with 100 µm sterile CellTrics (Partec, Münster, Germany). For morphological evaluation, recovered cells were plated in 4-multiwell chamber slides. After 24 hours cells were fixed and stained for Phalloidin and DAPI (Invitrogen) and analyzed though fluorescence inverted microscopy (Nikon, Tokyo, Japan).

### Senescence detection

Recovered cells and tumor sections embedded in optimum cutting temperature (OCT) were stained for β-Galactosidase using the senescence β-Galactosidase staining kit (Cell Signaling Technology, Beverly, Massachusetts, USA). Briefly slides were washed with PBS 1X and incubated with a fixative solution for 10–15 minutes. After washing with PBS 1X, slides were stained overnight with β-Galactosidase solution (final pH 6.0) at 37 °C in a dry incubator (slides were covered with parafilm to prevent staining solution evaporation). Pictures were taken with a Nikon Eclipse 80i microscope (Nikon). Direct senescence evaluation in MDA-MB-231 cultured within the scaffold, grown on chamber slides or in *in vivo* tumor tissue was performed by lipofuscin staining with Sudan Black B, as previously reported^54^.

### HIF-1 α and pimonidazole fluorescent staining

OCT embedded scaffolds and tumor sections were stained as follows: samples were fixed with cold acetone for 5 minutes, washed twice in PBS 1X and incubated over night with the anti HIF-1α antibody (1:500, Abcam) at 4 °C. Samples were then washed twice in PBS 1X and incubated for 90 minutes with the goat anti-rabbit phycoerythrin secondary antibody at room temperature (Invitrogen). Nuclei were counterstained with DAPI (Invitrogen). The pimonidazole staining was performed using the Hypoxyprobe Plus Kit (100 mg pimonidazole HCl plus 1 unit of 4.3.11.3 mouse FITC-MAb and one unit of anti-FITC HRP) (Hypoxyprobe, Burlington, MA, USA) according to manufacturer’s instructions.

### Flow cytometry

Cells in monolayer cultures or 3D scaffolds were harvested by trypsinization or by enzymatic digestion in Collagenase type I (Merck Millipore), respectively. To determine cell viability, cells were stained with 50 µM calcein AM and 2 mM ethidium homodimer-1 (Invitrogen). The TUNEL assay was performed using the *In-Situ* Cell Death Detection Kit (Roche, Basel, Switzerland) according to the manufacturer’s protocol. The cell suspensions were assayed using BD FACS CantoI (Beckmann Coulter, Brea, CA, USA).

### Immunohistochemical analysis

Immunohistochemistry was performed as previously described^53^. Briefly, scaffolds were dehydrated by incubation in increasing concentrations of ethanol (30–100%), embedded in paraffin, sliced with a rotating microtome (Leica Biosystems, Wetzlar, Germany) at 5 µM thickness and mounted to Superfrost Plus microslides (Thermo Fisher Scientific, Waltman, MA, USA). Hematoxylin and eosin staining was performed to evaluate scaffold architecture, cell morphology and distribution. For monolayer cultures, 100 000 cells were cytospinned onto glass slides and fixed for 10 minutes in acetone and 5 minutes in chloroform. The following antibodies were used for the staining according to the manufacturer’s instructions: anti cytokeratin AE1/AE3 (1:400, Abcam, Cambridge, UK); anti HIF-1α (1:500, Abcam) anti GLUT-1 (1:500, Abcam).

### Western blot

Proteins were isolated with a lysis buffer composed of 50 mM Tris-HCl (pH 8), 150 mM NaCl, 1% Triton X-100, 0.1% SDS and the Halt Protease and Phosphatase Inhibitor Cocktail (Thermo Fisher Scientific, Waltman, MA, USA). The BCA protein assay kit (Thermo Fisher Scientific) was used to quantify the protein content. An equal amount of protein from each sample was separated on CriterionTM Precast Gel Tris-HCl (Biorad, Hercules, CA, USA) and transferred to polyvinylidene fluoride membranes (Millipore Corporation, Billerica, MA, USA), as previously described^55^. The membranes were blocked for 2 hours in 5% non-fat dry milk PBS with 0.1% Tween 20 (Sigma-Aldrich) at room temperature and incubated overnight at 4 °C with primary antibody. After washing, the membranes were incubated for 1 hour at room temperature with horseradish-peroxidase-conjugated secondary antibody. The following primary antibodies were performed: anti- Collagen IV (1:1000, Abcam), anti-Fibronectin (1:1000, Abcam), anti-Vitronectin (1:1000, Abcam), anti-CASP3 (1:1 000, Cell Signaling Technology), anti-CASP9 (1:500, Cell Signaling Technology), anti-BAX (1:1000 Cell Signaling Technology), anti-RHO (1:1000 Merck Millipore), anti-Vinculin (1:1000 Thermo Fisher Scientific). Vinculin was used as the loading control.

### VEGF secretion

The secretion of VEGF was evaluated in MCF-7 and MDA-MB-231 culture media by ELISA kit (R&D systems, Minneapolis, MN, USA) according to the manufacturer’s instructions. In order to compare 2D and 3D conditions, the assessed VEGF concentrations were normalized against the number of cells for each time point.

### *In vivo* study

Female immunodeficient NU/NU nude mice [NU-Foxn1nu] 6 weeks old were purchased from Charles River Laboratories for subcutaneous xenograft experiments. Mice were maintained under pathogen free conditions and on low-fluorescence diet according to the guidelines set forth by the National Institutes of Health.

MDA-MB-231 and MCF-7 2 × 10^6^ cells marked with Luciferase probe were suspended in 100 µl PBS and orthotopically injected into the fat pad of the left inguinal mammary gland of each mouse (5 mice per experimental group). For the experiments using MCF-7 cells, a 60 day 0.72 mg slow release estradiol pellet (Innovative Research of America (IRA), Sarasota, FL, USA) was subcutaneously implanted in the back of the mice 24 hours prior to cells injection for estrogen supplementation. Tumor growth was followed by *in vivo* bioluminescence imaging (BLI) using the Xenogen IVIS 200 *in vivo* Bioluminescence Imaging System (PerkinElmer, Waltham, MA, USA) every 2–3 days after cell injection in 4 mice per each group, and tumor volume was assessed at each time point by caliper measurement in all mice. Immediately before starting the imaging procedure 100 mg/kg of D-Luciferin potassium substrate (PerkinElmer 1 g) was injected into the tail vein of each mouse, images were acquired 10 minutes after injection. Image acquisition was performed by a computer running Living Image software (Xenogen, Alameda, CA, USA). After 9 weeks tumors were collected and processed as follows: tumors were fragmented into small pieces and stored in TRizol at −80 °C for RNA extraction; stored in lysis buffer at −80 °C for protein extraction; embedded using the optimum cutting temperature (OCT) compound (VWR International, Radnor, PA, USA) in a cryomold and instantly frozen at −20 °C. Ten micron-thick slides were sectioned by cutting OCT tumor blocks with a cryostat and slides were stored at −20 °C.

### Statistics

Three independent replicates were performed for each experiment. Data are presented as mean ± standard deviation (S.D.), or mean ± standard error of the mean (S.E.M.), as stated, with n indicating the number of replicates. For *in vitro* and *in vivo* data, differences between groups were assessed by a two-tailed Student’s t-test and accepted as significant at *p* < 0.05. For patient samples data, differences between groups were assessed by the Two-sample Wilcoxon rank-sum (Mann-Whitney) test and accepted as significant at *p* < 0.01. Specificity and sensitivity were determined by receiver-operating characteristic (ROC) analysis.

### Study approval

The retrospective patient study was approved by the Ethical Committee of the Istituto Scientifico Romagnolo per lo Studio e la Cura dei Tumori (IRST) IRCCS (protocol number 993), and conducted in accordance with the Declaration of Helsinki. Written informed consent was obtained from patients before sampling. Experimental procedures on animals were performed according to the guidelines set forth by the National Institutes of Health. All procedures were reviewed and approved by the Institutional Animal Care and Use Committee (IACUC) of the Houston Methodist Research Institute (HMRI), protocol number AUP 0614–0033.

## Supplementary information

Supplementary Dataset 1

## Data Availability

The datasets used and analyzed during the current study are available from the corresponding author on reasonable request.
