# Supplementary material for: A biomimetic 3D model of hypoxia-driven cancer progression
Source: Sci Rep. 2019 Aug 22;9:12263. doi: 10.1038/s41598-019-48701-4 (PMC6706452; doi:10.1038/s41598-019-48701-4)
Supplement: Supplementary file 1 — Supplementary Dataset 1 [file 41598_2019_48701_MOESM1_ESM.pdf]

Supplementary Information

A biomimetic 3D model of hypoxia-driven cancer progression

Chiara Liverani[1], Alessandro De Vita[1], Silvia Minardi[2], Yibin Kang[3], Laura Mercatali[1], Dino Amadori[1], Alberto Bongiovanni[1], Federico La Manna[1], Toni Ibrahim[1,*,#] & Ennio Tasciotti[2,4,#]

[1]Osteoncology and Rare Tumors Center, Istituto Scientifico Romagnolo per lo Studio e la Cura dei Tumori (IRST) IRCCS, via P. Maroncelli 40, Meldola, Italy. [2]Center for Biomimetic Medicine, Houston Methodist Research Institute (HMRI), 6670 Bertner Ave. Houston, TX 77030, USA. [3]Department of Molecular Biology, Princeton University, Princeton, NJ 08544, USA. [4]Houston Methodist Orthopedics & Sports Medicine, Houston Methodist Hospital, Houston, TX 77030, USA.

[#]These authors contributed equally to this work.

[*]Correspondence and requests for materials should be addressed to T.I. (email: toni.ibrahim@irst.emr.it), Osteoncology and Rare Tumors Center, Istituto Scientifico Romagnolo per lo Studio e la Cura dei Tumori (IRST) IRCCS, via P. Maroncelli 40, 47014, Meldola, Italy. Tel.: +39 0543 739239, fax: +39 0543 739151.

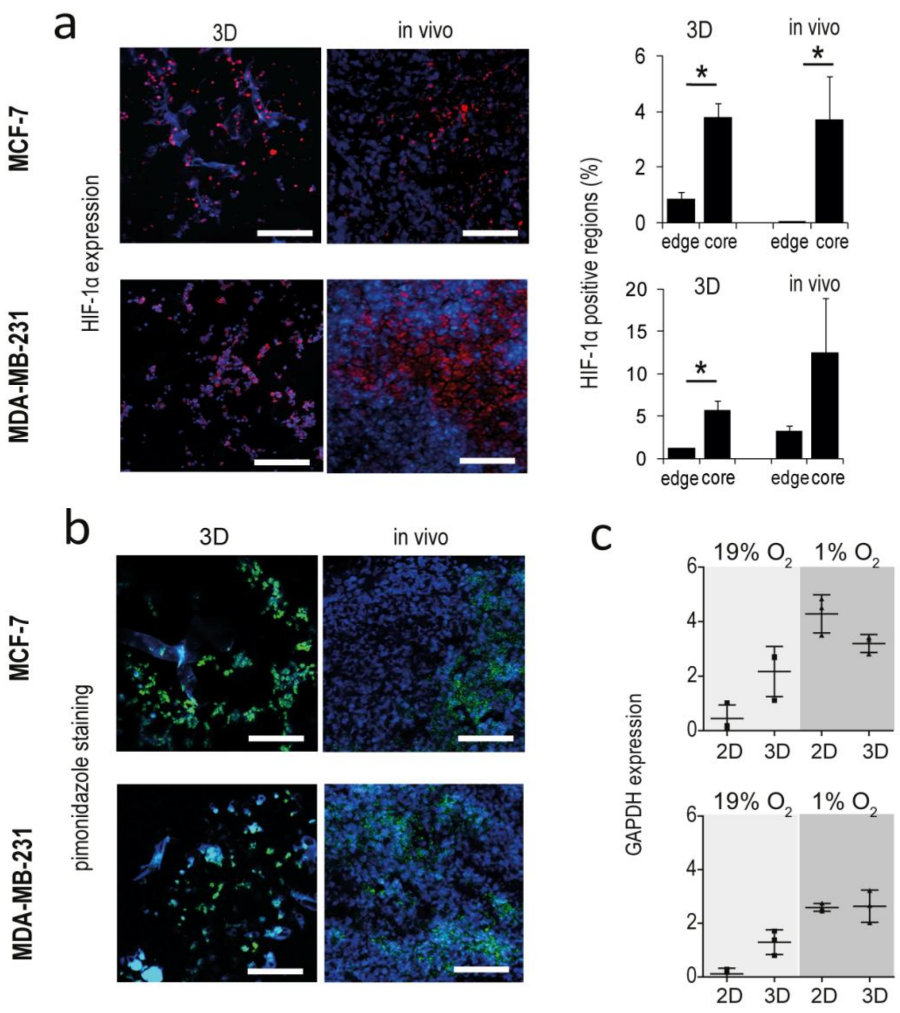

Supplementary Fig. S1

(a) Representative images of HIF-1α positive MCF-7 and MDA-MB-231 in histological sections of the scaffold (day 7) (3D) and of the *in vivo* tumors (*in vivo*) and percentages of positivity in core or edge regions of the sections. Scale bars: 100 µm. Data represent mean ± (S.D.). (n=3) *p<0.05, two-tailed Student's t-test. HIF-1α-positive cells (red), nuclei stained with DAPI (blue) and collagen scaffold autofluorescence (blue). **(b)** Representative images of pimonidazole-stained histological sections of MCF-7 and MDA-MB-231 within the scaffold (day 1) and in *in vivo* tumors. Pimonidazole-positive spots (green), nuclei stained with DAPI (blue) and collagen scaffold autofluorescence (blue). Scale bars: 100 µm. **(c)** Box-plot diagrams showing GAPDH expression levels in 2D or 3D cultured MCF-7 and MDA-MB-231 under normoxic or hypoxic states (19% O_2 or 1% O_2, respectively). Data represent mean ± S.D. (n=3).

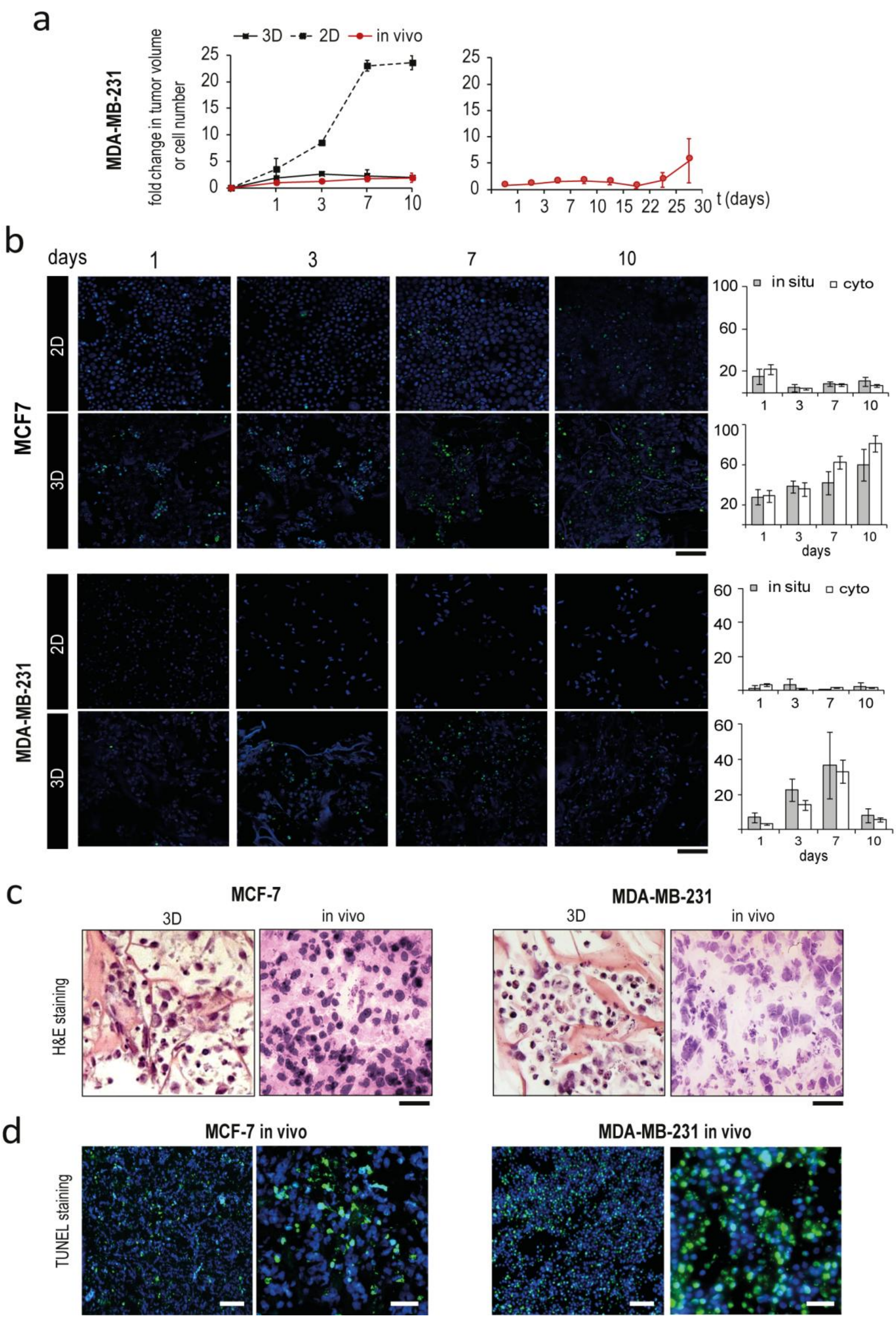

Supplementary Fig. S2

(a) Fold changes in cell number (relative to day 0) of MDA-MB-231 in monolayer culture (2D) within the 3D scaffold (3D), and fold changes in tumor volume (relative to

day 0) of MDA-MB-231 orthotopically implanted into a murine model (*in vivo*) on days 1, 3, 7 and 10, and complete *in vivo* growing curve. Data represent mean ± S.D. (n=3 for 2D and 3D data, and n=5 for *in vivo* data). **(b)** Representative images of MCF-7 and MDA-MB-231 apoptotic cells in TUNEL-stained 2D slides or scaffold sections (3D). TUNEL-positive cells (green) and nuclei stained with DAPI (blue), scale bars: 100 µm. Percentages of apoptotic cells detected by in-situ staining or flow cytometry (cyto) analysis. Data represent mean ± S.D. (n=3). **(c)** Representative images of MCF-7 and MDA-MB-231 with condensation or fragmentation of nuclei in hematoxylin and eosin-stained histological sections of the scaffold (day 7) and the corresponding *in vivo* tumors. Scale bars: 20 µm. **(d)** Representative images of MCF-7 and MDA-MB-231 apoptotic cells in TUNEL-stained *in vivo* tumor sections. TUNEL-positive cells (green) and nuclei stained with DAPI (blue). Left panels, scale bars: 200 µm. Right panels, scale bars: 50 µm.

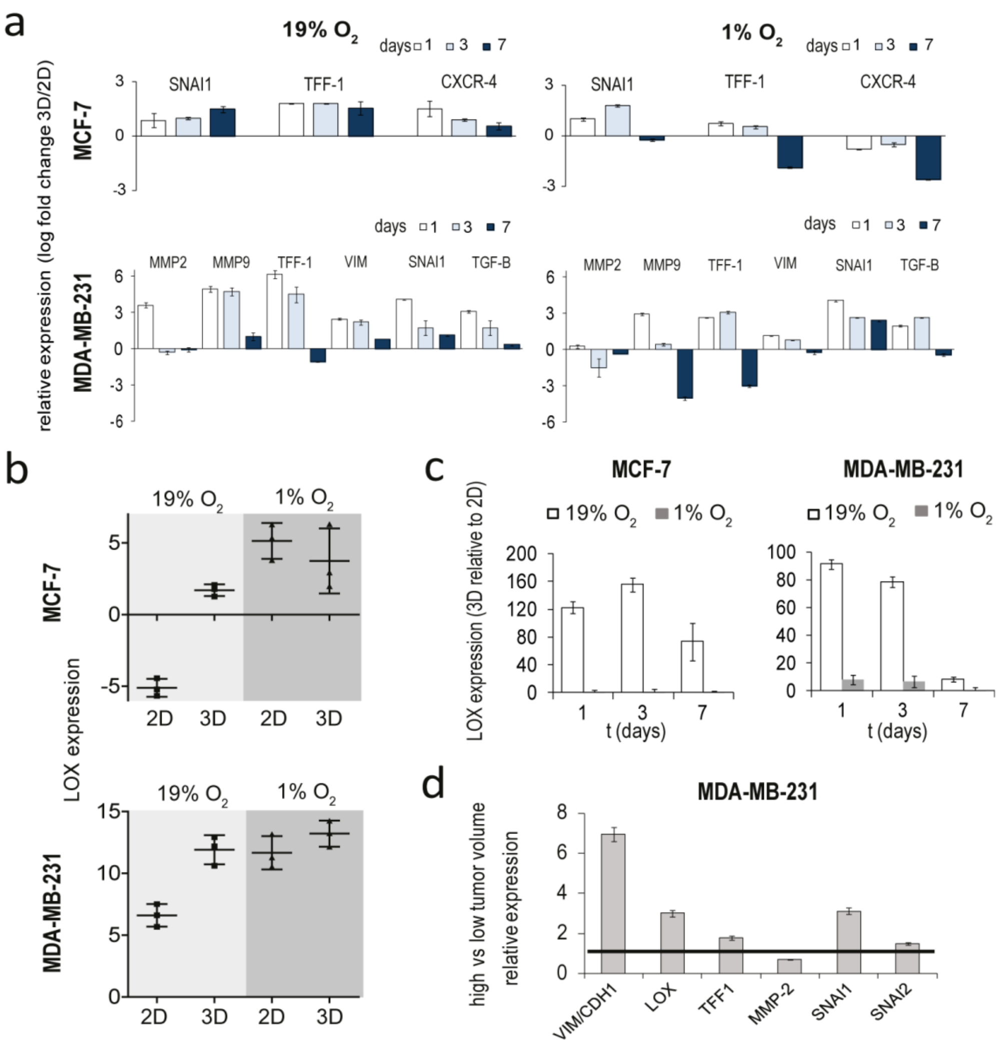

Supplementary Fig. S3

(a) Relative expression levels of SNAI1, TFF-1 and CXCR4 in MCF-7, and MMP2, MMP9, TFF-1, VIM, SNAI1 and TGF-β in MDA-MB-231 cultured within the 3D scaffold versus monolayer culture on days 1, 3 and 7 under normoxic or hypoxic states (19% O_2 or 1% O_2, respectively). Data represent mean ± S.D. (n=3). **(b)** Box-plot diagrams showing LOX expression levels in 2D or 3D cultured MCF-7 and MDA-MB-231 under normoxic or hypoxic states (19% O_2 or1% O_2, respectively). Data represent mean ± S.D. (n=3). **(c)** Relative expression levels of LOX in MCF-7 and MDA-MB-231 within the 3D scaffold vs. monolayer culture on days 1, 3 and 7 under normoxic or hypoxic states (19% O_2 or 1% O_2, respectively). **(d)** Relative expression values of biomarkers related to tumor aggressiveness in high vs. low volume MDA-MB-231 *in vivo* tumors. Data represent mean ± S.D. (n=3).

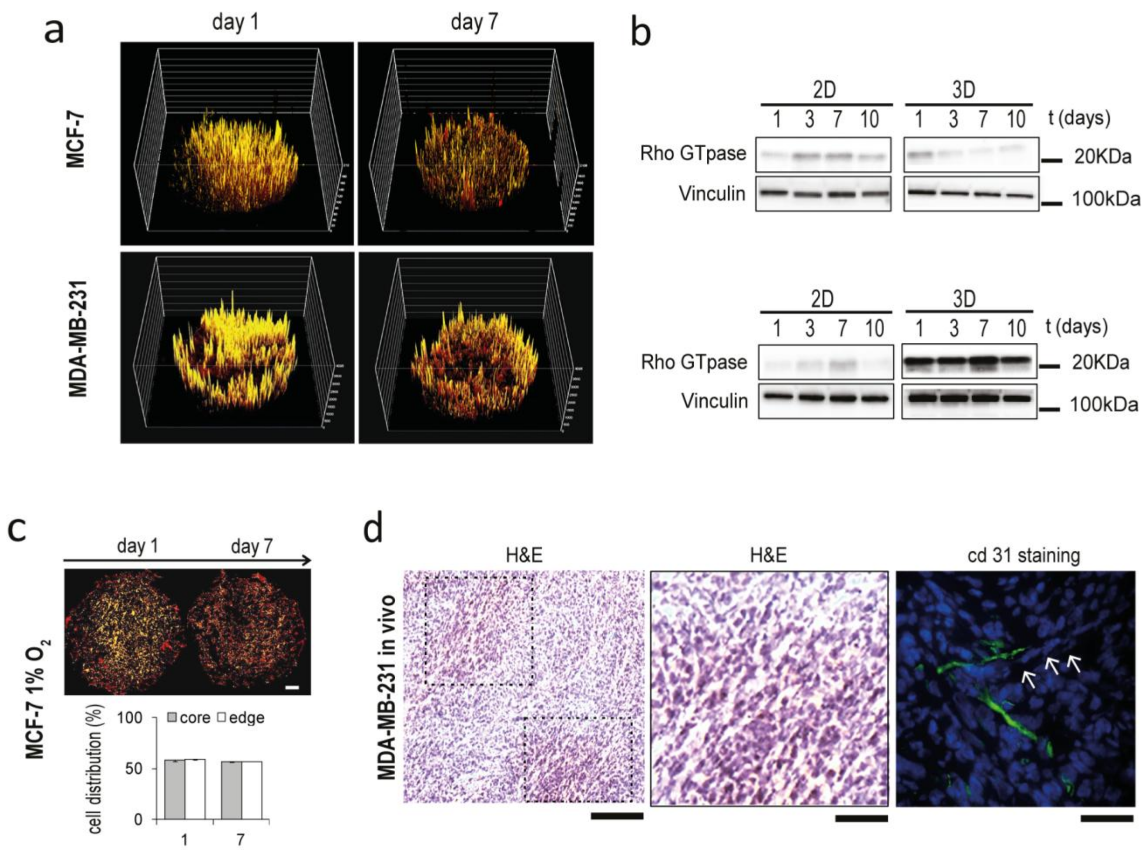

Supplementary Fig. S4

(a) Intensity plot of whole histological sections of the scaffold with MCF-7 and MDA-MB-231 on days 1 and 7. Cells are stained with DAPI (yellow), collagen scaffold autofluorescence (red). **(b)** Western blot for Rho GTPase (22kDa) in MCF-7 and MDA-MB-231 on days 1, 3, 7 and 10 in 2D and 3D cultures. **(c)** Whole images of histological sections of 3D cultured MCF-7 on days 1 and 7 under hypoxic state (1% O_2), and percentages of cells in edge or core regions of the scaffold. Scale bars: 1 mm. Data represent mean ± S.E.M. (n=3). Cells are stained with DAPI (yellow), and red is the collagen scaffold autofluorescence. **(d)** Representative images of cells in “Indian file” patterns (arrows) in hematoxylin-eosin and Cd31 stained histological sections of MDA-MB-231 *in vivo* tumors. Cd31-positive cells (green) and nuclei stained with DAPI (blue). Scale bars: 300, 100 and 50 µm.

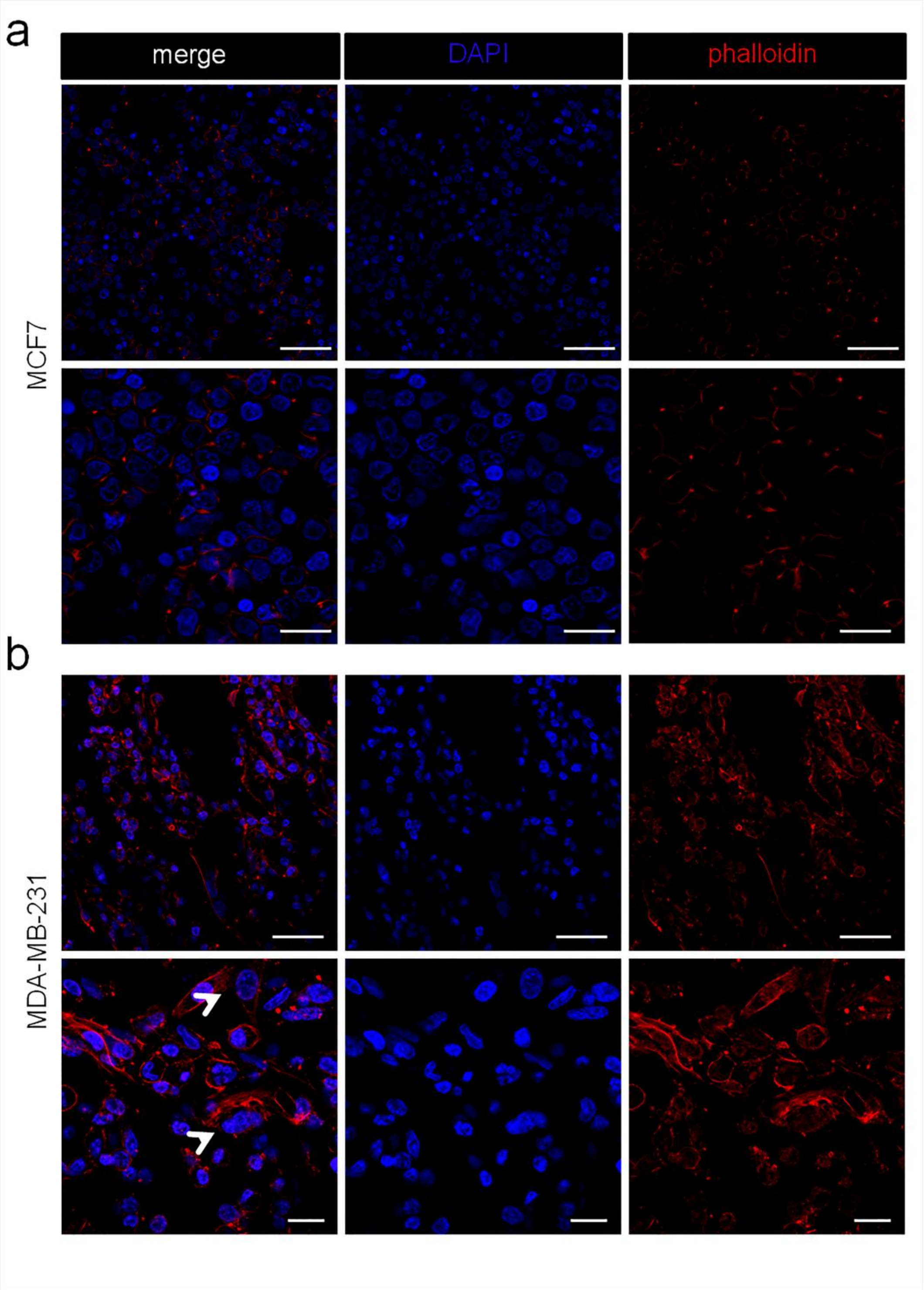

Supplementary Fig. S5.

Representative confocal microscopy images of **(a)** MCF-7 and **(b)** MDA-MB-231 within the scaffold on day 7. Phalloidin staining for F-actin (red) and DAPI-stained nuclei (blue). Scale bars: 50 µm in upper (a) and (b) panels, 20 µm in lower (a) and (b) panels.

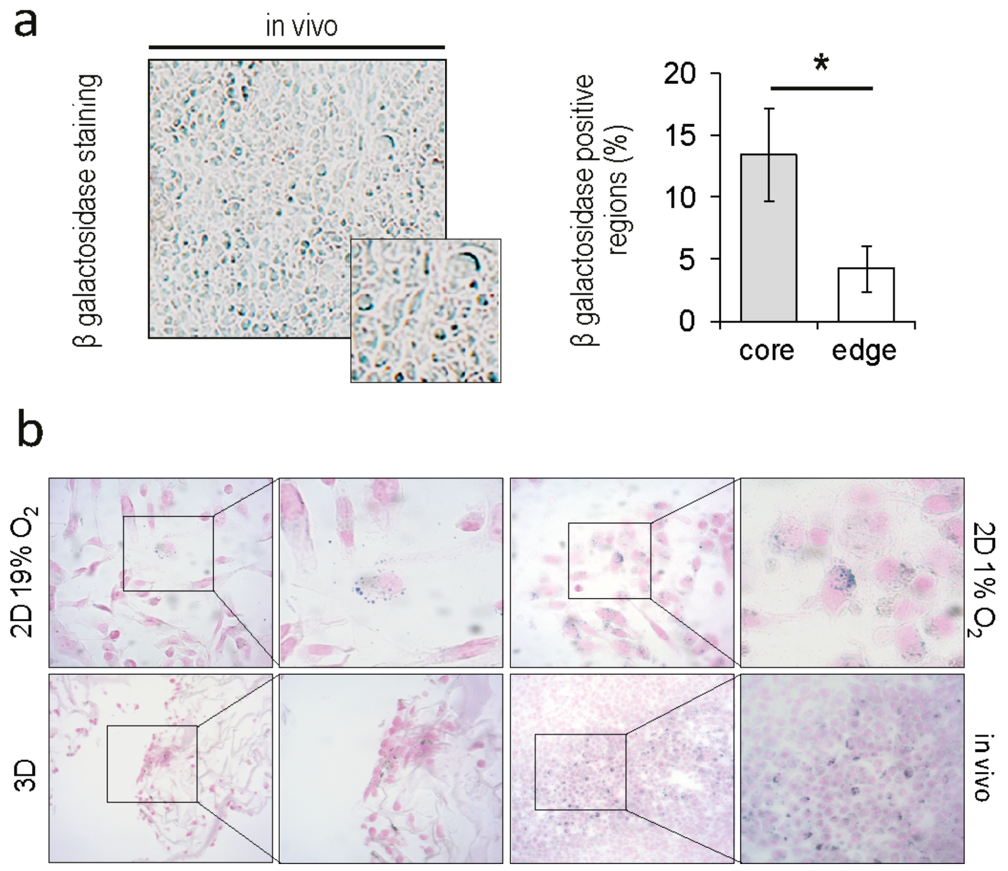

Supplementary Fig. S6.

(a) Representative images of cells positive for β-galactosidase staining (blue) in MDA-MB-231 *in vivo* tumors, and percentage of area positive for the β-galactosidase staining. **(b)** Representative images of cells positive for lipofuscin staining (blue) in MDA-MB-231 growing within the scaffold (3D), in monolayer culture under normoxic and hypoxic state (2D 19% O_2, 2D 1% O_2) or in *in vivo* tumors (*in vivo*). Nuclei were counterstained with 0.1% Nuclear Fast Red.

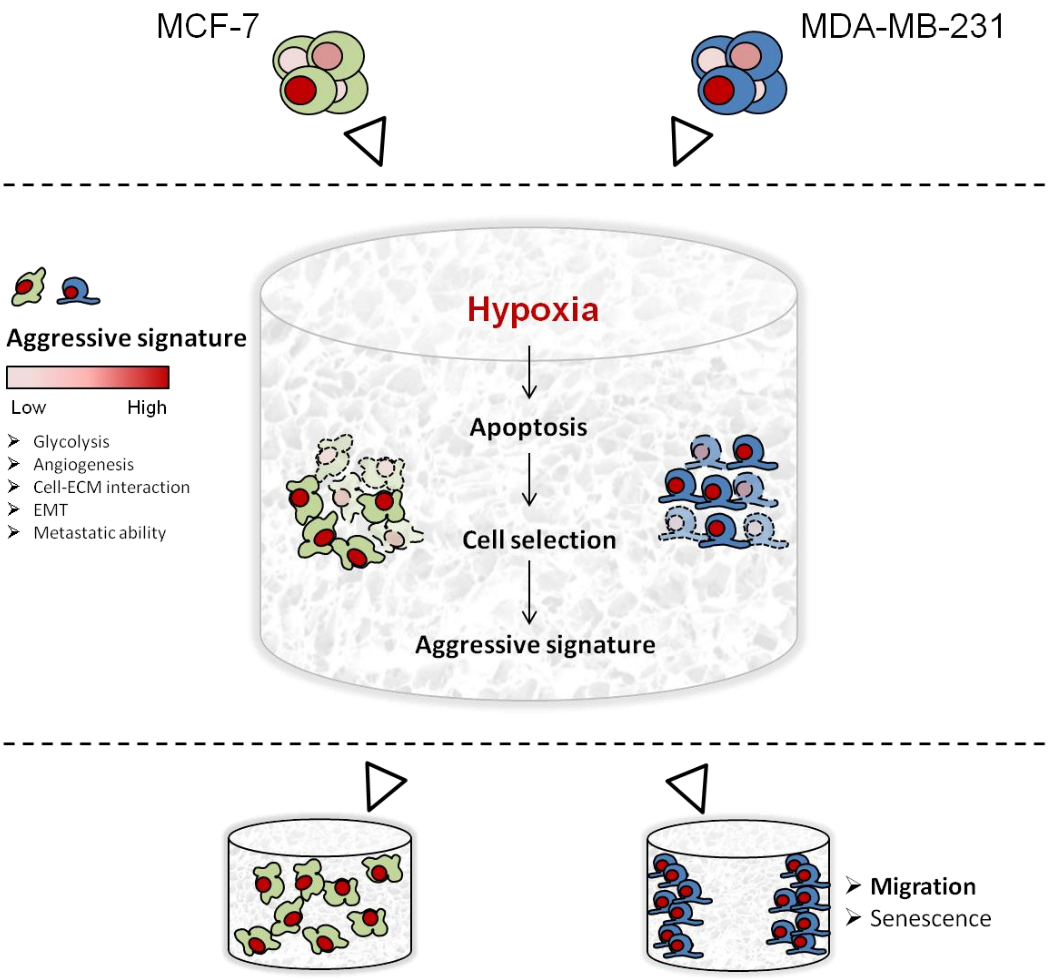

Supplementary Fig. S7

Schematic representation of the cancer cell behavior in biomimetic scaffolds. The scaffold models the evolution of solid tumors: the hypoxic niche induces apoptosis and selection of cancer cells with highly aggressive phenotype. Cells acquire a signature of genes and pathways related to glycolysis, angiogenesis, cell-matrix interaction, EMT, and metastatic ability. Mesenchymal-like MDA-MB-231 respond to hypoxia and restore viability by activating migration on the collagen fibrils and by undergoing cellular senescence. Epithelial MCF-7 do not exhibit these behaviors.

Supplementary Table S1.

Physical properties of collagen scaffolds.

	Average	SD
Scaffold volume (µm^3)	$57.7 \cdot 10^3$	± 4.5
Pore area (µm^2)	$24.7 \cdot 10^3$	± 1.3
Volume of collagen (µm^3)	$8.8 \cdot 10^3$	± 0.5
Void space (µm^3)	$48.9 \cdot 10^3$	± 0.2
Porosity (%)	84.8	± 6.3
Pore wall thickness (µm)	14.7	± 1.0

SD, standard deviation.
